# Serological Responses to Trachoma Antigens prior to the Start of Mass Drug Administration: Results from Population-Based Baseline Surveys, North Darfur, Sudan

**DOI:** 10.4269/ajtmh.23-0608

**Published:** 2024-03-19

**Authors:** Angelia M. Sanders, Balgesa E. Elshafie, Zeinab Abdalla, Courtney Simmons, Erica Brook Goodhew, Tania A. Gonzalez, Andrew W. Nute, Atif Mohammed, Elizabeth Kelly Callahan, Diana L. Martin, Scott D. Nash

**Affiliations:** 1 Trachoma Control Program, The Carter Center, Atlanta, Georgia; 2 Sudan Federal Ministry of Health, Khartoum, Sudan; 3 Trachoma Control Program, The Carter Center, Khartoum, Sudan; 4 United States Centers for Disease Control and Prevention, Atlanta, Georgia

## Abstract

After years of programmatic inaccessibility, in 2019–2020 the Sudan Federal Ministry of Health Trachoma Control Program conducted population-based trachoma surveys in three localities (districts) in North Darfur state, Sudan. These baseline surveys were to determine the prevalence of trachomatous inflammation–follicular (TF) among children aged 1–9 years and to further use serological markers to understand the historical trachoma burden within this mass drug administration (MDA)–naive area. Trained and certified graders collected trachoma clinical data, and trained nurses collected dried blood spot (DBS) samples. The DBSs were assayed on a multiplex bead array for antibody responses to the *Chlamydia trachomatis* antigens Pgp3 and CT694. Across the three localities, 3,613 individuals aged 1–9 years and 3,542 individuals aged ≥15 years were examined for clinical signs, and 8,322 DBSs were collected. The prevalence of TF among children aged 1–9 years was endemic (≥5%) in two localities (El Seraif, 15.6%, and Saraf Omrah, 11.0%) and below the TF elimination threshold (<5%) in the third (Kotom, 1.4%). The Pgp3 seroprevalence among children aged 1–9 years was 34.1% in El Seraif, 35.0% in Saraf Omrah, and 11.0% in Kotom. Locality prevalence results were similar for Pgp3 and CT694. Seroprevalence increased with age in all three localities. Serological data collected within these surveys demonstrate that all three localities have had a long history of exposure to *Chlamydia trachomatis* and that two of the three localities require MDA to reach elimination as a public health problem threshold.

## INTRODUCTION

Trachoma is caused by the bacterium *Chlamydia trachomatis* and is a public health problem in 23 countries within Africa.^1,2^ Ocular *C. trachomatis* infection is self-limiting, but repeated infections can lead to conjunctival scarring that over time can cause the upper eyelid to turn in (trachomatous trichiasis [TT]) and the eyelashes to rub against the eyeball to the point of blindness due to corneal opacity.^1,3^ National trachoma programs use the prevalence of clinical signs of trachomatous inflammation–follicular (TF) among children aged 1–9 years and TT among those aged ≥15 years to determine the need for implementation of the WHO-endorsed Surgery, Antibiotics, Facial cleanliness, and Environmental improvement (SAFE) strategy.^1^ If TF prevalence is ≥5%, then one or more rounds of annual community-wide mass drug administration (MDA) with antibiotics is recommended to help drive down infection. Once TF is <5% and TT is <0.2% in all enumeration units (districts or their equivalent), a country can be validated as having eliminated trachoma as a public health problem.^1^

The WHO recommends that national trachoma programs conduct a population-based prevalence survey for both mapping (at baseline) and impact assessment after MDA to estimate the district-level prevalence of TF among children aged 1–9 years.^4^ Recently, trachoma control programs serving trachoma-endemic countries have been incorporating serological monitoring into their surveys as a complementary trachoma indicator.^5–7^ Population-level serological responses have correlated well with TF at the district level and thus present programs with an opportunity to monitor trachoma with a more objective indicator.^5,8^ Monitoring TF requires ensuring that field graders can grade clinical manifestations reliably over the course of a survey, and TF has been continually shown to overestimate actual *C. trachomatis* infection once MDA programs have begun.^9,10^ To better understand the utility of sero-surveillance in post-MDA settings, more data are needed from populations that have yet to receive MDA for trachoma. Owing to the success of the global trachoma program and the scale-up of MDA as warranted, few of these areas still exist.

Sudan is endemic for trachoma.^11–13^ As a result of periods of insecurity in the Darfur region of Sudan, there are multiple localities (administrative units for healthcare delivery) in this region that have yet to have baseline mapping conducted and therefore have yet to begin implementation of the SAFE strategy in areas that are suspected of being endemic. The Sudan National Trachoma Control Program conducted baseline surveys in three localities in North Darfur state that incorporated the collection of dried blood spots (DBSs) from all examined individuals of all ages. The aim of this study was to generate baseline data on trachoma clinical signs for decision-making around the need to implement the SAFE strategy and to obtain locality-wide seroprevalence data to determine trachoma transmission intensity in MDA-naive populations.

## MATERIALS AND METHODS

### Survey design.

**FIGURE 1.**
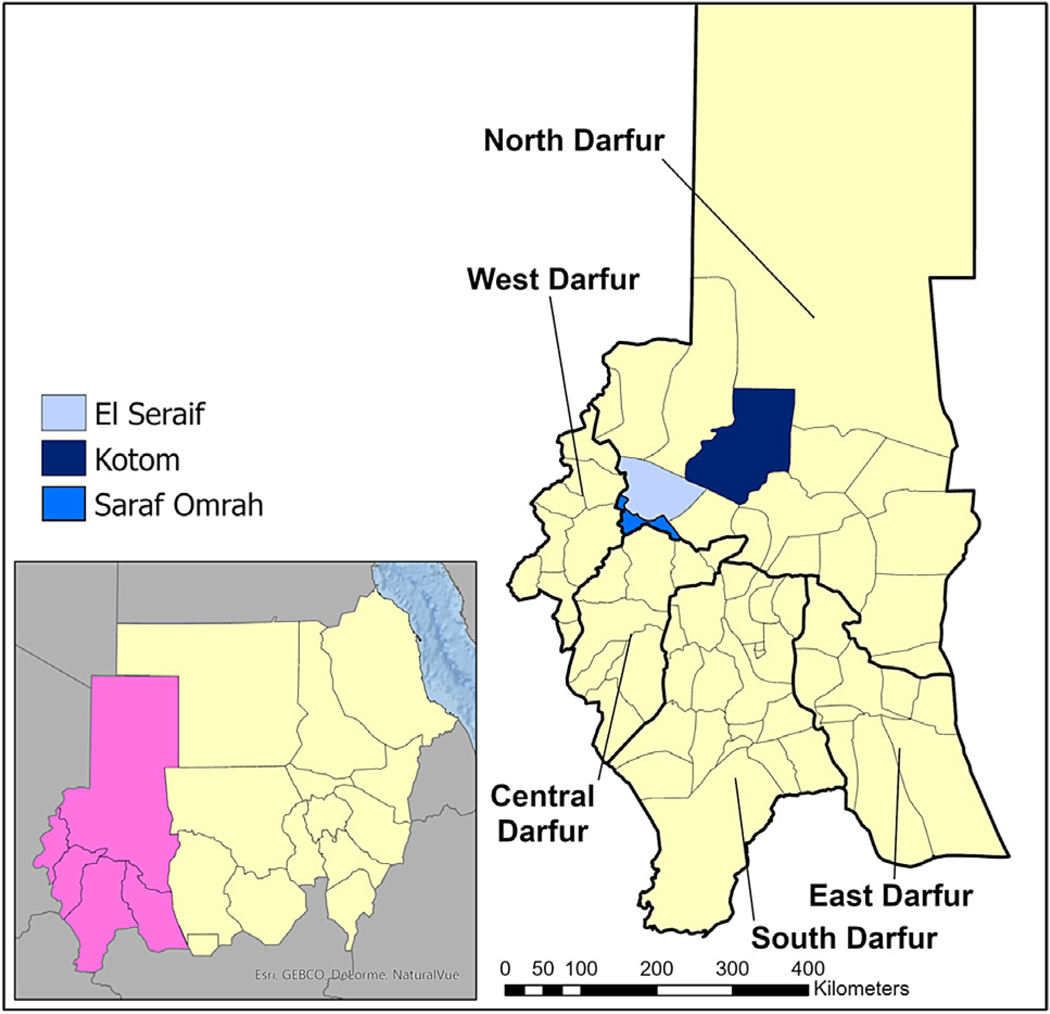
Location of the three surveyed localities within North Darfur, Sudan, 2019–2020.

Between November 2019 and January 2020, population-based surveys were conducted in three localities in North Darfur state: El Seraif, Kotom, and Saraf Omrah (Figure 1). No trachoma interventions had taken place in these localities prior to the baseline surveys.

To estimate a TF prevalence of 10% among children aged 1–9 years with a precision of 3%, given a design effect of 2.65 and a 95% confidence interval (CI), a total sample size of 1,018 children was needed.^14^ Assuming a nonresponse rate of 20% yielded a target population size of 1,222 children per locality, we further assumed 4.7 individuals per household and that children aged 1–9 years made up 35% of the population. Based on these assumptions and targeted sample size, we surveyed 30 clusters (villages) and within each cluster surveyed 25 households.

A two-stage procedure was used to select the sample. In the first stage, clusters were selected randomly from a geographically ordered list provided by the State Ministry of Health Expanded Program on Immunization. Villages with populations <250 and >5,000 persons (urban centers) were not included for logistical reasons.^11^ In addition, an internally displaced persons (IDPs) camp with a population of more than 28,000 residents in Kotom locality was excluded. In the second stage, village community leaders created a list of households by household father’s name, and individual households were grouped into five-household “segments” and numbered. The numbers were written on individual pieces of paper and placed in a bowl for random selection by a village leader. Five segments were chosen per cluster.

### Data collection.

#### Training of data collectors.

Each survey team was composed of a trachoma grader, DBS collector, and data recorder. Trachoma “graders” were Sudanese ophthalmologists and ophthalmic medical assistants whose training consisted of in-class and field practice using the WHO-simplified grading system.^15^ Each grader was required to pass an in-class slide test that included all five stages of trachoma and a field reliability examination with a ≥0.70 kappa score against the consensus grade of the grader trainers to join the survey teams. The graders completed their training in 2019. Dried blood spot collectors were certified nurses living in the Darfur region who underwent a 2-day training on sterile technique and DBS collection and handling. Data collectors underwent a 2-day training on how to collect data electronically using cellular phones to document consent, conduct structured household interviews, and scan DBS barcodes. All data recorders were required to pass an examination on their data collection skills to participate on the survey team. After the training, a pilot survey was conducted in one village in a neighboring locality not included in the survey sampling frame, with each team practicing the survey process and DBS collection.

#### Household questionnaire.

A structured household interview was conducted in Arabic with an adult household respondent at each selected household, with special preference given to female caregivers. The interview included questions regarding demographics, socioeconomic indicators, time to collect water (<30 minutes, 30–60 minutes, and >60 minutes), face washing practices of children, and access to a latrine.^16^ Data collectors visually verified if a latrine was present. After the interview, all residents of selected households were enumerated, regardless of their presence and/or willingness to be clinically examined for trachoma or participate in blood collection.

#### Clinical examination.

Clinical examination was conducted by certified trachoma graders using a 2.5× loupe and a flashlight if needed. All present and consented children aged 1–9 years were examined for TF, trachomatous inflammation–intense (TI), and TT as defined by the WHO simplified grading scheme. Children were also assessed for a clean face, defined as the absence of both ocular and nasal discharge. All participants aged ≥1 year were examined for TT. Any participant found to have TF or TI was provided antibiotics, together with members of their family, in accordance with national guidelines. If TT was observed, the individual was registered and counseled to have TT surgery during the next scheduled surgical campaign in their locality.

#### Dried blood spot collection.

Dried blood spot collectors used a retractable lancet to collect finger prick blood onto a filter paper (TropBio Pty Ltd., Townsville, Australia) containing six extensions calibrated to hold 10 μL of blood. All consenting household members aged ≥1 year had a finger pricked, and their blood (60 μL) was collected on filter paper. Filter papers were labeled with a barcode, scanned into the survey software, air-dried for at least 2 hours, and stored in Ziploc bags with desiccant at −20°C until flown at ambient temperature to be tested at the Centers for Disease Control and Prevention (CDC) in Atlanta, GA.

#### Multiplex bead assay.

One bloodspot extension for each person, corresponding to 10 μL of whole blood (5 *μ*L of sera), was eluted overnight at 4°C and diluted to a final sera concentration of 1:400 in Buffer B (1× phosphate-buffered saline [PBS], 0.5% casein, 0.5% polyvinyl alcohol, 0.8% polyvinylpyrrolidone, 0.3% Tween-20, and 0.02% NaN_3_) containing 3 μg/mL *Escherichia coli* extract to block nonspecific binding.

Antigen-coupled beads were added to 96-well filter-bottom plates (Millipore, Bedford, MA) and washed twice with 0.05% Tween-20 in PBS (PBST). Control sera and bloodspot eluates (1:400) were then added, beads suspended, protected from light, and plates shaken at room temperature for 1.5 hours. After beads were washed three times with 100 μL PBST, total IgG was detected with 50 ng of biotinylated mouse anti-human total IgG (clone H2; Southern Biotech, Birmingham, AL) and 40 ng of biotinylated mouse anti-human IgG4 (clone HP6025; Invitrogen, South San Francisco, CA) per well in 50 μL assay buffer (1× PBS, 0.5% bovine serum albumin, 0.02% NaN_3_, and 0.05% Tween-20). After a second wash step, streptavidin-phycoerythrin (SAPE; Invitrogen, South San Francisco, CA) was added at a concentration of 250 ng per well in assay buffer and incubated for 30 minutes at room temperature. After washing to remove SAPE, any loosely bound antibodies were removed with an additional incubation in assay buffer. After a final wash in PBST, beads were suspended in 100 μL PBS and stored overnight at 4°C. The following day, plates were shaken and read on a Luminex instrument (Luminex Corp., Austin, TX) equipped with Bio-Plex Manager 6.0 software (Bio-Rad, Hercules, CA). The median fluorescence intensity minus background (MFI-BG) thresholds for Pgp3 and CT694 were 850 and 146, respectively.

## STATISTICAL ANALYSES

All data were collected electronically on cellular phones loaded with the custom-built survey software NEMO.^11^ Sampling weights were calculated as the inverse of the probability of selection at both stages of sampling. Confidence intervals were calculated using Taylor linearization through *survey* v. 4.1–1 in R v. 4.0.2. Weighted estimates accounted for the multilevel structure of survey sampling. Poststratification weighting using 5-year age-sex bands from the survey census population was used when estimating the prevalence of TT among the whole population and among those aged ≥15 years. Trachomatous trichiasis unknown to the health system was defined as anyone who had not had surgery and had not refused surgery for at least one eye presenting with TT.

Age-specific seroprevalence curves were constructed for each locality surveyed. Logistic regression was used to test for associations between age, sex, and TF, adjusting for clustering at household and village levels. We estimated *C. trachomatis* force of infection with the seroconversion rate (SCR) to Pgp3 and CT694 among children aged 1–9 years from age-structured seroprevalence using a generalized linear model with a complementary log–log link and robust standard errors.^17,18^ The model assumed stationarity (constant force of infection) and no seroreversion.^5^

The SCR was further estimated for all ages, using two distinct serocatalytic models as previously described.^19,20^ Briefly, both models used a Bayesian Markov chain Monte Carlo method to estimate seroconversion (λ) and seroreversion (*ρ*) rates using age as a proxy for time. Model 1 assumed a constant age-dependent rate of seroconversion and seroreversion. Model 2 assumed a time point of change in which the SCR (λ) significantly and substantially decreased. Model 2 attempted to estimate a historic SCR (λ0) prior to this time point of change, the proportional decline in transmission (γ), seroreversion rate (*ρ*), and SCR after the time point of change (λ1). All-age model diagnostics were compared for each antigen and locality via the fit plots of autocorrelation, deviance information criteria, Gelman-Rubin statistic, and effective sample size to determine the best fitting model. Previously published informative priors for γ and *ρ* were additionally applied to Model 2 for all districts.^20^ The best fitting models for each locality were determined. All statistical analyses were performed in R Studio v. 4.0.2 (RStudio, PBC, Boston, MA).

## RESULTS

Across the three surveyed localities, 10,121 individuals were enumerated, 3,924 (39%) of whom were children aged 1–9 years and 4,562 (45%) of whom were individuals aged ≥15 years (Table 1). Of enumerated children, 3,613 (92%) were examined for clinical signs and 3,674 (94%) had a DBS sample taken. Among individuals aged ≥15 years, 3,542 (78%) were examined for TT and 3,534 (77%) provided a DBS sample.

Indicators of water, sanitation, and hygiene varied across the three localities (Supplemental Table 1). Household latrine prevalence ranged from 31.1% (95% CI: 17.4–49.0%) in Saraf Omrah to 63.4% (95% CI: 46.8–77.3%) in Kotom. Households within 30 minutes roundtrip of a water source ranged from 34.9% (95% CI: 20.0–53.4%) in Kotom to 76.5% (95% CI: 61.6–86.9%) in Saraf Omrah. Clean faces among children aged 1–9 years ranged between 63.9% (95% CI: 45.4–79.0%) in El Seraif to 76.2% (95% CI: 69.5–81.9%) in Saraf Omrah.

The prevalence of TF among children aged 1–9 years was 15.6% (95% CI: 10.9–21.7%) in El Seraif, 1.4% (95% CI: 0.8–2.7%) in Kotom, and 11.0% (95% CI: 7.6–15.7%) in Saraf Omrah (Table 2). Trachomatous inflammation–intense was ≤5% in all three localities. The TF prevalence was lower in females than in males (*t* = −2.18, *P* = 0.038) in El Seraif, but it was not substantially different in the other two localities. Within this age group, TF point estimates were higher among younger children and decreased with age; however, this trend was only statistically significant among children in Saraf Omrah (*t* = −3.35, *P* = 0.002) (Supplemental Figure 1). The prevalence of TT among adults aged ≥15 years ranged from 0.4% (95% CI: 0.1–1.2%) in El Seraif to 0.8% (95% CI: 0.4–1.8%) in Saraf Omrah. The prevalence of TT unknown to the health system was similar to the prevalence of TT.

**FIGURE 2.**
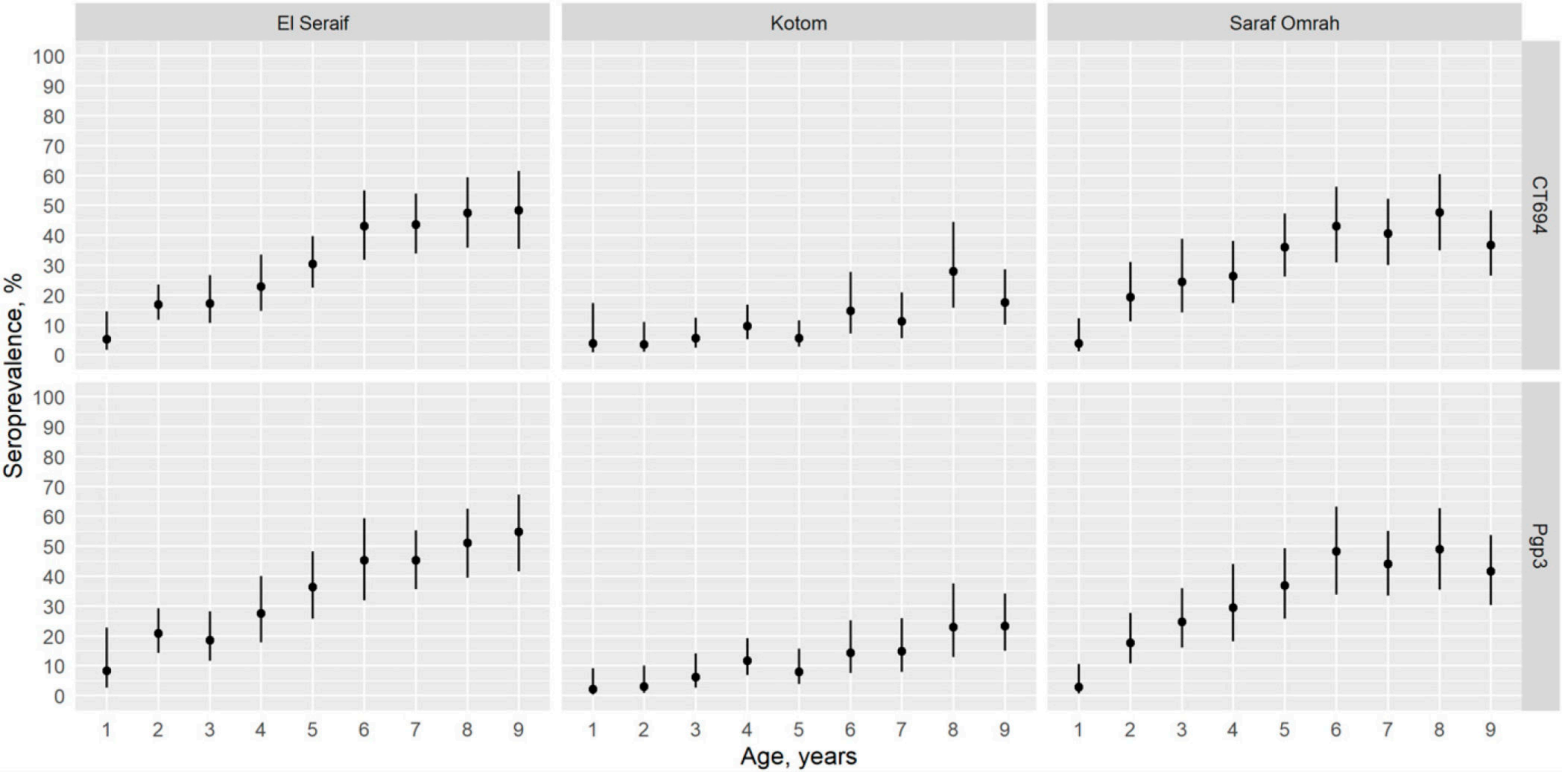
Age-specific seroprevalence of CT694 (top panels) and Pgp3 (bottom panels) among children aged 1–9 years within the three surveyed localities in North Darfur, Sudan, 2019–2020. Bars represent 95% confidence intervals.

**FIGURE 3.**
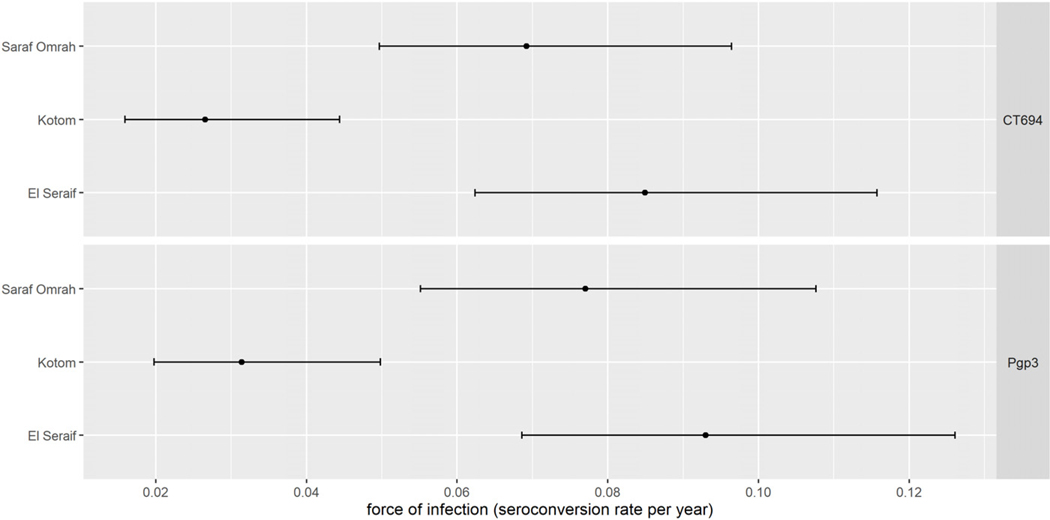
Seroconversion rate among children aged 1–9 years within the three surveyed localities in North Darfur, Sudan, 2019–2020. Bars represent 95% confidence intervals.

The prevalence of antibody responses to Pgp3 among children aged 1–9 years ranged from 11.0% (95% CI: 6.7–17.7%) in Kotom to 35.0% (95% CI: 27.4–43.3%) in Saraf Omrah, whereas the prevalence of CT694 ranged from 10.3% (95% CI: 6.0–17.2%) in Kotom to 32.9% (95% CI: 25.3–41.6%) in Saraf Omrah. Clusters with a high prevalence of Pgp3 (≥45%) were clustered in the south of Saraf Omrah bordering South Darfur (Supplemental Figure 2). Seropositivity by year of age among children across the three localities is shown in Figure 2. The SCR for Pgp3 was 0.03 (95% CI: 0.02–0.05) seroconversions per child-year in Kotom, 0.08 (95% CI: 0.06–0.11) in Saraf Omrah, and 0.09 (95% CI: 0.07–0.13) in El Seraif (Figure 3). The SCR for CT694 was of similar magnitude to that of Pgp3 for all three localities.

**FIGURE 4.**
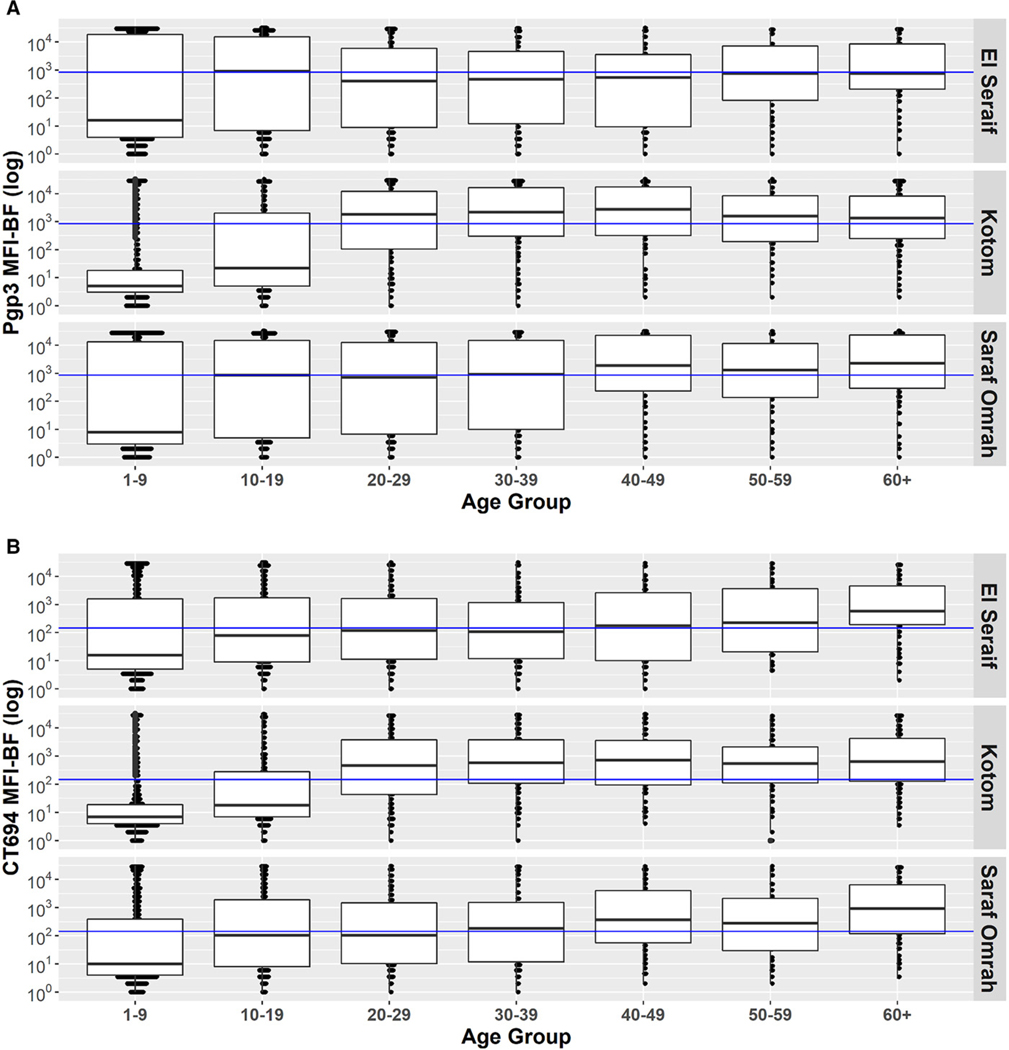
Distribution of antibody responses to **A**) Pgp3 and **B**) CT694 by age group within the three surveyed localities in North Darfur, Sudan, 2019–2020. Blue line signifies the positive threshold. MFI-BF = median fluorescence intensity minus background. Boxes represent the median and the 25th and 75th quartiles. Bars represent the range.

**FIGURE 5.**
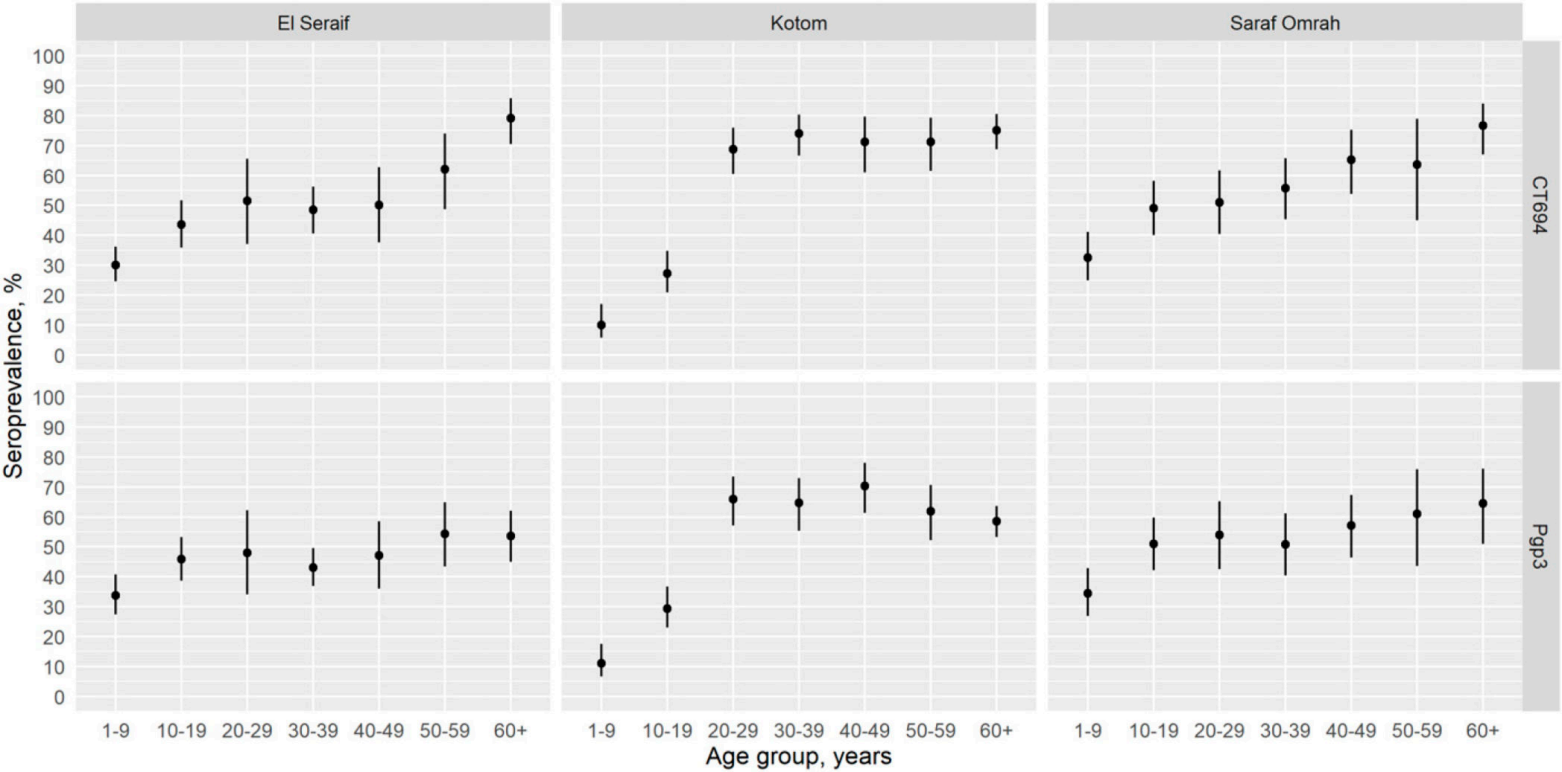
Age-specific seroprevalence of CT694 (top panels) and Pgp3 (bottom panels) among participants by age group in the three surveyed localities in North Darfur, Sudan, 2019–2020. Bars represent 95% confidence intervals.

The intensity of antibody responses (MFI-BG) among children aged 1–9 years was considerably lower in Kotom than in the other two localities, whereas the intensity of responses was similar across the three localities starting with the 20–29-year age group (Figure 4). Similarly, age group–specific seroprevalence was lower in Kotom through age group 10–19 years but similar to the other localities by ages 20–29 years (Figure 5). Seroprevalence in Kotom reached 66.5% (95% CI: 57.6–74.4%) and 69.3% (95% CI: 60.8–76.7%) by age group 20–29 years for Pgp3 and CT694, respectively. After serocatalytic models were applied to the all-age data for each locality (Supplemental Table 2), a single SCR model, assuming a constant SCR over time (Model 1), was the best fit to characterize the seroprevalence trends across all ages in all three localities, including Kotom (Table 3).

## DISCUSSION

The data from these surveys indicated that two of the three localities had a TF prevalence above the elimination threshold; thus, WHO guidelines recommend A, F, and E interventions. Although the serology data from children aged 1–9 years align with the TF prevalence data, the all-age serology data suggest that all three of these North Darfur localities have had a long history of exposure to trachoma. Moreover, the TT data from adults are above the elimination threshold in all three localities. More investigation is needed to understand why Kotom has a current TF prevalence below threshold in the absence of MDA or other interventions, especially considering that the serology and TT data suggest a history of trachoma in this locality.

In Sudan, trachoma is no longer a public health problem in most previously endemic localities.^11,21^ Of the localities still requiring SAFE interventions, the majority are in the Darfur region and have a TF <20%.^13^ The Darfur region has a history of major armed conflict spanning multiple decades, which led to the displacement of millions of Darfuris between 2003 and 2013.^22^ This history of insecurity has frequently prevented the completion of baseline surveys and delayed the implementation of SAFE activities. Between 2014 and early 2023, there was a period of relative stability, and thus these baseline surveys were conducted to help the Program get closer to completing baseline mapping. The mass movement of people has also made it challenging to identify and measure the magnitude of trachoma among all populations at risk within the country. The Program in Sudan has a history of trachoma monitoring and implementation within refugee and IDP camps.^12,13,23^ In White Nile State, for example, it was demonstrated that refugee camps were endemic for trachoma, whereas the surrounding localities were not.^12^ Kassab IDP camp in Kotom locality was excluded from the sampling frame for the Kotom survey given its size of over 28,000 residents. Given the results of these surveys, it would be helpful if the Trachoma Control Program could conduct a separate survey in Kassab IDP camp to understand the disease burden in this marginalized community and provide interventions if required.

This study used standard trachoma surveys to provide important pre-MDA data on serological responses to trachoma antigens. To date, few surveys or studies have collected serological data from MDA-naive evaluation units with moderate to high levels of TF. In an international meeting of trachoma experts in 2019, the collection of serological data from baseline pre-MDA settings was considered of primary importance for the global trachoma program.^24^ Pre-intervention serology data from districts that are clearly trachoma endemic, such as El Seraif and Saraf Omrah (TF: 15.6%, 11.0%) in North Darfur, will be important in setting benchmarks for how age-seroprevalence curves are impacted through the course of trachoma elimination efforts (impact surveys, surveillance surveys, and post-elimination monitoring). We can see the importance of looking at serology in pre-MDA settings by comparing these data with those from the neighboring Amhara region, Ethiopia. In 2017, Dera district in Ethiopia had a similar TF prevalence (14.7%) to these two localities, but the Pgp3 seroprevalence was considerably lower (11.3% after 11 rounds of MDA) compared with El Seraif (34.1%) and Saraf Omrah (35.0%).^5^ In fact, seroprevalence for El Seraif and Saraf Omrah was closer to that of Andabet district in Amhara (Pgp3 = 36.9%), which was considered a hyperendemic district (TF = 37.0%). These findings could be evidence of the effect of MDA over many years suppressing antibody seroconversion among children in Amhara. As trachoma control efforts progress in these two localities, data from repeat serosurveys will help to better understand the relationship between TF and seroprevalence and to inform the Program on the sensitivity of serology as a program monitoring tool.

Survey teams collected DBSs from all individuals aged ≥1 year to determine the cumulative exposure to *C. trachomatis* in this population over time. In all three surveyed localities, seroprevalence increased with age over the age span, reaching a seroprevalence of ≥50% among adults. This is clear evidence that trachoma transmission has been occurring over the long term in this part of North Darfur, even in Kotom where TF and seroprevalence were much lower than in the other localities. In accordance with this historical exposure, the prevalence of TT among individuals ≥15 years in each locality was well above the threshold for the elimination of trachoma as a public health problem. Although the seroprevalence was equally high among adults in all three localities, the seroprevalence among individuals younger than 20 years in Kotom was well below that of the other two localities. Accordingly, the TF prevalence among children in Kotom was <5% despite the locality having similar WASH indices as the other two localities and no history of trachoma interventions. Although models did not detect a statistically significant change in transmission intensity among age groups in Kotom, it is possible that environmental or social conditions experienced by children in Kotom are different from those experienced by older adults in that locality or by children in the other two localities.

Based on these survey results and current trachoma antibiotic treatment recommendations, El Seraif and Saraf Omrah localities are eligible for three or more annual rounds of MDA. In addition, more investment in water, sanitation, and hygiene programs is recommended for these localities. For example, only Kotom had >50% of its population with access to a household latrine. After 3 years of A, F, and E interventions in El Seraif and Saraf Omrah, it will be important for the Program to collect DBSs again to evaluate the effect of multiple years of interventions on the seroprevalence among young children. The prevalence of TT observed in the three localities and the fact that the prevalence of TT and TT unknown to the health system were almost identical, highlight the fact that little has been done to reduce the levels of TT in these communities. Investments are needed to train TT surgeons and to conduct TT surgical campaigns to prevent blindness and reduce the prevalence of TT to below the elimination threshold.

This study had some limitations. Because of cold-chain concerns, conjunctival swabbing to estimate the prevalence of ocular *C. trachomatis* infection was not conducted as part of these surveys. Although conjunctival swabbing is not part of standard trachoma surveys, infection data would have helped provide more information on the relationship between TF, infection, and serological responses in treatment-naive populations. Use of sample collection media that preserve DNA/RNA at ambient temperatures should be considered for future work in remote areas such as the survey area. The serological assay used in this study could not discriminate between ocular and urogenital *C. trachomatis* infection; however, for those samples taken from children aged 1–9 years, it was unlikely that there was considerable misclassification. Among adults, exposure to urogenital *C. trachomatis* cannot be ruled out. Among those aged ≥20 years, Pgp3 prevalence reached ≥50%, suggesting that antibody positivity would likely be due in large part to exposure to ocular transmission, as such high rates of sexually transmitted infections within the general population are rarely seen.^25^ For purposes of incorporating DBSs into routine survey activities, it is worth noting that this approach required an additional team member and training, as well as costs related to the filter paper and serological analysis.

## CONCLUSIONS

The Trachoma Control Program in Sudan used the opportunity of baseline surveys in suspected trachoma endemic areas of the Darfur region to collect serological data during population-based surveys. These baseline data in intervention-naive communities, followed by years of MDA interventions and impact surveys incorporating DBS collection, could help national and global trachoma programs better determine whether serological monitoring could be a programmatic tool for trachoma control. This approach should be considered in other countries that have yet to complete baseline mapping in suspected trachoma endemic areas and in areas where the ability to conduct neglected tropical disease data collection activities is limited owing to access and insecurity.

## Supplementary Material

Supplementary Material

## Figures and Tables

**TABLE 1:** Sample size for the three surveyed localities in North Darfur, Sudan, 2019–2020

Sample Size Category	El Seraif	Kotom	Saraf Omrah
Households enumerated	740	876	729
Households consented and examined	655	805	660
Children aged 1–9 years enumerated	1,359	1,242	1,323
Children aged 1–9 years examined	1,251	1,105	1,257
Individuals aged ≥15 years enumerated	1,505	1,771	1,286
Individuals aged ≥15 years examined	1,148	1,322	1,072
Children aged 1–9 years providing DBSs	1,275	1,127	1,272
Individuals aged ≥15 years providing DBSs	1,148	1,320	1,066
Individuals, all ages, providing DBSs	2,848	2,781	2,693

DBS = dried blood spot.

**TABLE 2:** Prevalence of TF, TI, TT, and antibody response to trachoma antigens Pgp3 and CT694 within the three surveyed localities in North Darfur, Sudan, 2019–2020

Trachoma Indicator	El Seraif	Kotom	Saraf Omrah
TF, ages 1–9 years	15.6% (10.9–21.7)	1.4% (0.8–2.7)	11.0% (7.6–15.7)
TI, ages 1–9 years	0.5% (0.2–1.7)	0.2% (0.1–1.1)	0.1% (0.0–0.8)
TT, ages ≥15 years	0.4% (0.1–1.2)	0.8% (0.4–1.5)	0.8% (0.4–1.8)
TT, ages ≥15 years “unknown” to health system	0.4% (0.1–1.2)	0.7% (0.4–1.4)	0.8% (0.4–1.8)
Pgp3 prevalence, ages 1–9 years	34.1% (27.7–41.1)	11.0% (6.7–17.7)	35.0% (27.4–43.3)
CT694 prevalence, ages 1–9 years	30.3% (24.8–36.5)	10.3% (6.0–17.2)	32.9% (25.3–41.6)

TF = trachomatous inflammation–follicular; TI = trachomatous inflammation–intense; TT = trachomatous trichiasis.

**TABLE 3:** Seroconversion rate among individuals aged 1–9 years and individuals of all ages within the three surveyed localities in North Darfur, Sudan, 2019–2020

District	Antigen	Static SCR 1–9 Years (95% CI)	Model*	SCR All Ages (before point of change) (2.5%, 97.5% IQR)	DIC
El Seraif	Pgp3	0.09 (0.07–0.13)	1	15.0 (14.1–18.0)	3,735.4
	CT694	0.09 (0.06–0.12)	1	11.3 (10.8–13.3)	3,700.5
Kotom	Pgp3	0.03 (0.02–0.05)	1	3.9 (3.8–4.4)	3,053.9
	CT694	0.03 (0.02–0.04)	1	3.4 (3.3–3.8)	2,845.4
Saraf Omrah	Pgp3	0.08 (0.06–0.11)	1	10.5 (9.9–12.3)	3,482.3
	CT694	0.07 (0.05–0.10)	1	3.4 (3.3–3.8)	2,845.4

DIC = deviance information criterion; IQR = interquartile range; SCR = seroconversion rate.

* Model 1 serocatalytic model assuming steady seroconversion rate.
