# Supplementary material for: Serological Responses to Trachoma Antigens prior to the Start of Mass Drug Administration: Results from Population-Based Baseline Surveys, North Darfur, Sudan
Source: Am J Trop Med Hyg. 2024 Mar 19;111(3 Suppl):49–57. doi: 10.4269/ajtmh.23-0608 (PMC11374501; doi:10.4269/ajtmh.23-0608)
Supplement: Supplemental Materials [file tpmd230608.SD1.pdf]

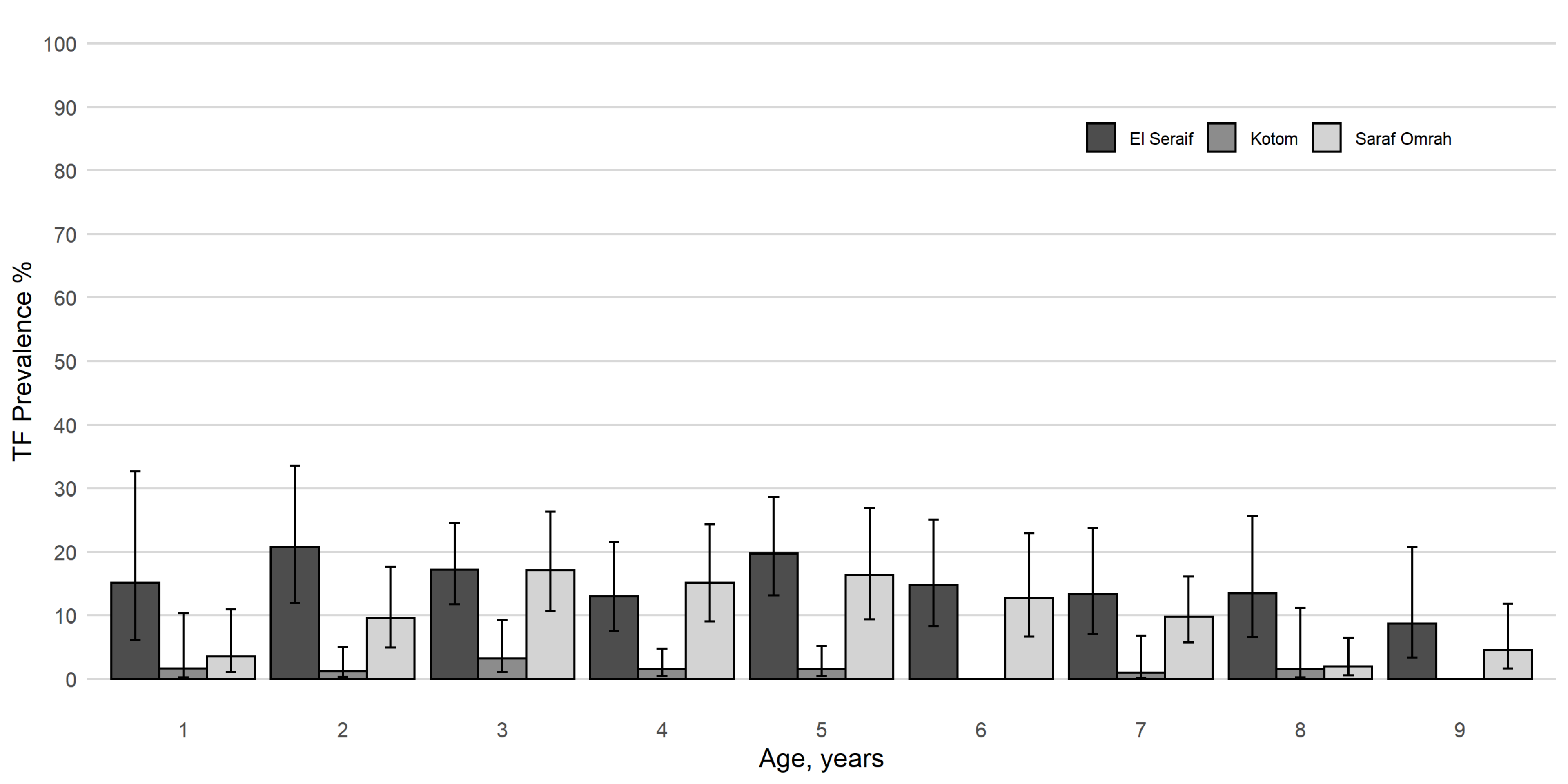

El Seraif
Kotom
Saraf Omrah
TF Prevalence %
100
90
80
70
60
50
40
30
20
10
0
1
2
3
4
5
6
7
8
9
Age, years

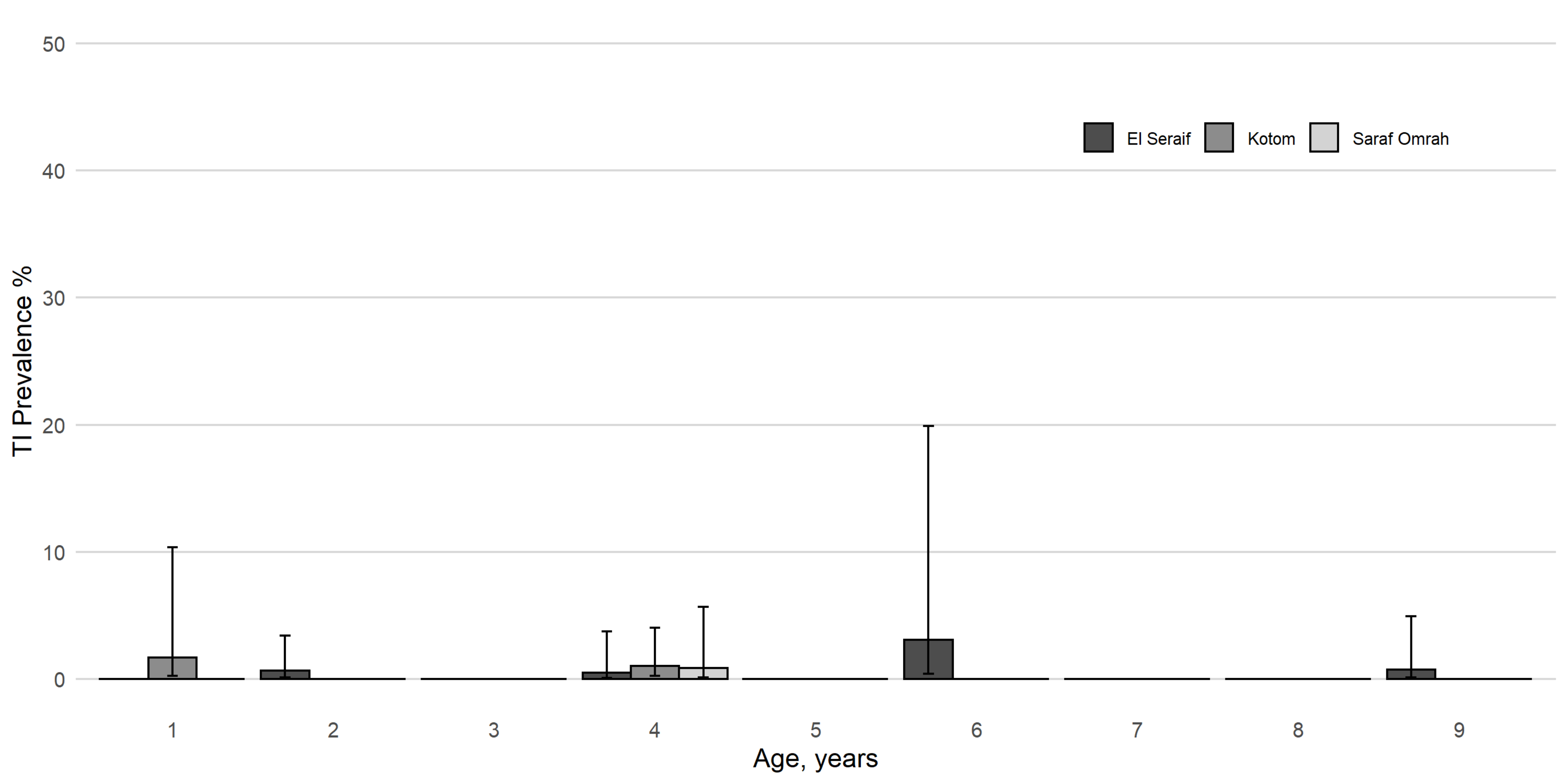
El Seraif
Kotom
Saraf Omrah
TI Prevalence %
50
40
30
20
10
0
1
2
3
4
5
6
7
8
9
Age, years

Supplemental Table 2. Full model diagnostics for the serocatalytic models within the 3 localities in North Darfur, Sudan, 2019-2020.

District	Antigen	Model	λ_0 (95%CrI)	GR	ESS	γ (95%CrI)	GR	ESS	ρ(95%CrI)	GR	ESS	λ_1 (95%CrI)	T_c	GR	ESS	LL	DIC
El Seraif	**Pgp3**	**1***	**15.0 (14.1, 18.0)**	**1.0**	**5127**	-	-	-	**17.5 (16.2, 22.1)**	**1.0**	**5186**	-	-	-	-	**9107.9**	**3735.4**
	**CT694**	**1***	**11.3 (10.8, 13.3)**	**1.0**	**5437**	-	-	-	**10.3 (9.5, 12.9)**	**1.0**	**5062**	-	-	-	-	**9482.6**	**3700.5**
	Pgp3	2	79.4 (64.7, 107.3)	1.6	18	15.5 (10.1, 29.2)	1.3	41	21.6 (18.7, 25.9)	1.1	69	0.12 (0.10, 0.15)	0.8 (0.7, 1.1)	1.0	19	696.4	3714.9
	CT694	2	64.8 (53.7, 83.9)	5.4	7	16.4 (11.5, 41.7)	3.2	13	17.0 (13.4, 19.7)	5.0	22	0.10 (0.08, 0.13)	0.7 (0.5, 0.8)	7.1	14	2182.5	3689.3
	Pgp3	2-ip	141.0 (103.2, 370.8)	3.1	16	1.9 (5.0, 10.4)	1.1	86	4.2 (4.7, 5.2)	1.0	651	0.07 (0.06, 0.08)	1.4 (1.0, 3.7)	12.0	589	2084.4	3848.1
	CT694	2-ip	6.0 (5.9, 6.6)	2.0	1366	12.5 (0.4, 97.4)	1.3	4	2.7 (2.4, 3.0)	1.0	792	0.01(0.00, 0.06)	0.06 (0.05, 0.07)	8.6	125	2903.3	3802.6
Kotom	**Pgp3**	**1***	**3.9 (3.8, 4.4)**	**1.0**	**3639**	-	-	-	**2.1 (1.9, 2.6)**	**1.0**	**4094**	-	-	-	-	**8544.5**	**3053.9**
	**CT694**	**1***	**3.4 (3.3, 3.8)**	**1.0**	**1777**	-	-	-	**0.8 (0.7, 1.1)**	**1.0**	**1777**	-	-	-	-	**7558.6**	**2845.4**
	Pgp3	2	39.4 (24.4, 130.2)	1.5	48	9.2 (2.8, 27.9)	1.2	47	3.8 (3.1, 4.5)	1.0	392	0.04 (0.03, 0.05)	0.4 (0.2, 1.3)	1.1	202	1715.2	2953.6
	CT694	2	23.3 (16.9, 82.0)	1.3	72	12.4 (3.7, 26.9)	1.2	136	2.2 (1.8, 2.7)	1.1	572	0.035 (0.031, 0.039)	0.23 (0.17, 0.82)	1.1	282	3668.2	2774
	Pgp3	2-ip	62.4 (44.7, 217.0)	2.9	15	5.6 (1.6 (11.5)	1.0	141	3.3 (2.9, 3.7)	3.3	811	0.03 (0.02, 0.04)	0.6 (0.5, 2.2)	12.8	385	4435.1	2977
	CT694	2-ip	15.5 (12.5, 38.4)	1.1	80	18.0 (7.3, 30.2)	1.0	204	1.9 (1.6, 2.2)	1.0	728	0.03 (0.02, 0.04)	0.2 (0.1, 0.4)	1.0	411	4462.2	2792.8
Seraf Omrah	**Pgp3**	**1***	**10.5 (9.9, 12.3)**	**1.0**	**5195**	-	-	-	**9.4 (8.6, 11.9)**	**1.0**	**4513**	-	-	-	-	**9272.8**	**3482.3**
	**CT694**	**1***	**3.4 (3.3, 3.8)**	**1.0**	**4009**	-	-	-	**0.8 (0.7, 1.1)**	**1.0**	**5347**	-	-	-	-	**8422.8**	**2845.4**
	Pgp3	2	26.0 (10.3, 223.1)	1.5	12	40.0 (4.7, 93.1)	1.7	4	9.5 (7.6, 17.0)	1.5	78	0.10 (0.09, 0.12)	0.3 (0.1, 2.2)	1.7	137	1847.5	2999.0
	CT694	2	16.2 (10.1, 85.6)	2.1	19	52.4 (9.8, 80.2)	2.6	5	6.3 (4.8, 8.3)	1.0	306	0.08 (0.07, 0.10)	0.16 (0.10, 0.86)	1.2	232	1748.8	3289.5
	Pgp3	2-ip	133.9 (100.4, 260.9)	3.0	32	5.1 (2.6, 10.5)	1.1	114	3.9 (3.4, 4.3)	1.0	789	0.07 (0.06, 0.08)	1.3 (1.0, 2.6)	7.9	475	2439.5	3509.5
	CT694	2-ip	8.5 (6.3, 29.4)	1.7	76	66.8 (19.2, 99.6)	1.4	47	2.4 (2.1, 2.7)	1.0	1041	0.06 (0.05, 0.07)	0.08 (0.06, 0.30)	3.1	66	3071.9	3413.6

*- best fitting model for each antigen for each district; λ0- estimated seroconversion - Model 1, estimated seroconversion rate prior to the timepoint of change - Model 2; γ- proportional decline in transmission over time; ρ- seroreversion rate; λ1- seroconversion rate after timepoint of change for Model 2; TC- timepoint of change (years ago) where significant seroconversion rate decline was detected; GR- Gelman-Rubin statistic; ESS- effective sample size; LL- log-likelihood; DIC- deviance information criteria.; -ip- indicates that a previously published informative prior for γ and/or ρ was applied to this model.
